# Systematic critical appraisal of GRADE recommendations for prostate cancer staging

**DOI:** 10.7705/biomedica.7653

**Published:** 2025-05-30

**Authors:** Dayana Sáenz, Ana Marcela Torres, Rodrigo Pardo, Wilfredo Donoso

**Affiliations:** 1 Unidad de Urología, Departamento de Cirugía, Facultad de Medicina, Universidad Nacional de Colombia, Bogotá, D. C., Colombia Universidad Nacional de Colombia Universidad Nacional de Colombia Bogotá, D. C. Colombia; 2 Grupo de Investigación e Innovación en Urología, Universidad Nacional de Colombia, Bogotá, D. C., Colombia Universidad Nacional de Colombia Universidad Nacional de Colombia Bogotá, D. C. Colombia; 3 Instituto de Investigaciones Clínicas, Facultad de Medicina, Universidad Nacional de Colombia, Bogotá, D. C., Colombia Universidad Nacional de Colombia Universidad Nacional de Colombia Bogotá, D. C. Colombia

**Keywords:** Prostatic neoplasms, neoplasm staging, practice guideline, grade approach, positron emission tomography/computed tomography, prostate-specific antigen, neoplasias de la próstata, estadificación de neoplasias, guía de práctica clínica, tomografía computarizada por tomografía de emisión de positrones, antígeno prostático específico

## Abstract

**Introduction.:**

Prostate cancer staging is necessary to determine tumor extent. In recent years, new and more accurate imaging modalities that could provide a better framework for patient management have emerged. They are currently incorporated into the prostate cancer guideline recommendations. Clinical practice guidelines are important for implementing clinical research findings and high-quality evidence-based recommendations.

**Objective.:**

To review and evaluate the quality of evidence underpinning the categorization of prostate cancer staging guidelines using the AGREE II tool.

**Materials and methods.:**

Systematic searches were performed on the PubMed, BiGG, and Epistemonikos databases. In addition, repositories and clinical practice guidelines websites were hand searched to identify GRADE recommendations for prostate cancer staging published in the last five years. The quality of clinical practice guidelines was assessed using the AGREE II tool. Recommendations and the certainty of evidence were also summarized.

**Results.:**

Seven guidelines that met the selection criteria were included. A narrative analysis of the staging recommendations and evidence mapping was performed. The AGREE II domain “clarity of presentation” had the highest score (mean = 71.59%), whereas “applicability” had the lowest score (mean = 45.15%). Five guidelines met the proposed AGREE II cutoff scores and provided staging and diagnostic recommendations.

**Conclusions.:**

Significant heterogeneity was observed in the methodological quality of the guidelines included, along with common deficits regarding applicability and stakeholder involvement. Thus, more rigorous and high-quality guidelines need to be developed to facilitate their implementation by clinicians in daily practice.

## Resumen

**Introducción.:**

La estadificación del cáncer de próstata es necesaria para determinar la extensión tumoral. En los últimos años, han surgido modalidades de imagen nuevas y más precisas que han mejorado el manejo de los pacientes. Las guías de práctica clínica son una herramienta importante para implementar los hallazgos de la investigación clínica basada en evidencia de alta calidad.

**Objetivo.:**

Revisar y evaluar la calidad de la evidencia de las guías de estadificación del cáncer de próstata mediante la herramienta AGREE II.

**Materiales y métodos.:**

Se realizó una búsqueda sistemática en las bases de datos PubMed, BiGG y Epistemonikos. También, se exploraron repositorios y sitios web de guías de práctica clínica para identificar recomendaciones GRADE de estadificación del cáncer de próstata publicadas en los últimos cinco años. La calidad metodológica de las guías se evaluó utilizando la herramienta AGREE II. Se resumieron las recomendaciones y la certeza de la evidencia.

**Resultados.:**

Siete guías cumplieron con los criterios de inclusión. Se hizo un análisis narrativo de las recomendaciones y un mapeo de la evidencia. El dominio “claridad de presentación” tuvo la puntuación más alta (media = 71,59 %), mientras que “aplicabilidad” tuvo la puntuación más baja (media = 45,15 %). Cinco guías cumplieron con el punto corte propuesto y contenían recomendaciones de estadificación y diagnóstico.

**Conclusiones.:**

Se observó una heterogeneidad significativa en la calidad metodológica de las guías incluidas; se encontraron falencias comunes relacionadas con la aplicabilidad y la participación de las partes interesadas.

Prostate cancer is the second most commonly diagnosed cancer among the male population ^1^. The American Cancer Society predicted approximately 288,300 prostate cancer cases in the United States for 2023 ^2^. In Colombia for 2022, the age-standardized rate incidence was 52.6 cases per 100,000, and the mortality rate was 12.2 cases per 100,000 ^3^. The likelihood of developing this type of cancer increases with age, peaking at 70% by the time individuals reach 80 years old. Early identification and treatment of prostate cancer is the most favorable prospect for a successful cure ^4^.

Risk group classifications consolidate clinical data regarding tumor extent, grade of pathology, and prostate-specific antigen (PSA) levels, all inherent indicators of tumor aggressiveness ^5^^,^^6^. Patients are recommended to undergo imaging studies to evaluate the local spread of the tumor as well as the involvement of lymph nodes, bones, and other organs. These assessments can be performed using traditional contrast abdominopelvic computed tomography or magnetic resonance imaging, complemented by chest radiography or computed tomography scans ^7^. Recent advancements in imaging techniques have introduced new modalities for staging high-risk patients, including prostate-specific membrane antigen (PSMA) positron emission tomography/computed tomography (PET-CT) ^7^. Enhanced precision in clinical risk stratification has the potential to establish a more effective framework for managing patients with prostate cancer.

Optimal stratification and management of prostate cancer remain to be a highly debated topic within the medical community. The Institute of Medicine defines clinical practice guidelines as “statements that provide recommendations for the optimization of patient care based on a systematic review of the evidence and an assessment of the benefits and harms of alternative care options” ^8^. Establishing clear criteria to evaluate the creation and reliability of strong recommendations is crucial for improving clinical decision-making. This strategy can be achieved by using standardized instruments such as the Appraisal of Guidelines for Research & Evaluation (AGREE) II and the Grading of Recommendations, Assessment, Development, and Evaluations (GRADE) approach ^9^^-^^12^. Prostate cancer guidelines were created to help healthcare providers determine the most effective evidence-based treatment approach for each patient, considering disease stage, risk level, age, life expectancy, and patient preferences. This study aimed to systematically review and evaluate the quality of evidence underpinning the categorization of clinical practice guidelines for prostate cancer using the AGREE II tool.

## Materials and methods

A systematic review was conducted according to the established methodologies proposed by the PRISMA statement ^13^. The PICOT format included population (patients with prostate cancer), intervention (staging for diagnosis of nodal, bone, and visceral metastases), comparison (does not apply), outcome (appraisal of the quality of evidence), and type of study (clinical practice guidelines).

### Search strategy

We conducted a methodical exploration to identify prostate cancer guidelines published within the past five years. We searched various electronic databases, such as PubMed, BiGG (the international database of GRADE guidelines), and Epistemonikos. In addition, supplementary data were obtained by hand searching guideline repositories and websites, including (but not limited to) the U.S. Preventive Services Task Force, GuiaSalud, MAGICapp, Chile Ministry Clinical Guidelines, International Agency for Research on Cancer publications, and the World Health Organization. The search strategy involved a combination of medical subject headings (MeSH) and unrestricted text terms (e.g., prostate, cancer, prostatic neoplasms, prostate cancer, GRADE approach or assessment, guidelines, clinical practice guidelines). One author performed the extraction process, which was cross-checked for consistency by another author who resolved the disagreements according to his judgment as the most experienced reviewer. For more information about data extraction, refer to the appendix (second section). The extracted data encompassed the characteristics of clinical practice guidelines and staging recommendations.

### Selection criteria

The eligibility criteria were: 1) Clinical guidelines related to prostate cancer, 2) Recommendations for staging, 3) Guidelines employing the GRADE approach, and 4) Publications from 2019 to 2023 to ensure inclusion of the most recent evidence-based guidelines. It is crucial to acknowledge the dynamic nature of prostate cancer research. Thus, outdated recommendations were excluded. Translations, summaries, interpretations, and draft guidelines were also excluded. No language constraints were imposed.

### Study selection

One reviewer conducted an initial record screening based on titles and abstracts and retrieved full-text guidelines for those potentially pertinent. A second independent reviewer validated the selection process using the same methodology. We applied the inclusion criteria and manually eliminated any duplicate records. The data were depicted in a Preferred Reporting Items for Systematic Reviews and Meta-Analysis (PRISMA) diagram ^13^.

### Data extraction

Two authors performed data screening and extraction based on the complete published versions of clinical practice guidelines and their respective supporting documents. Previous versions of those clinical practice guidelines were also considered. One author performed the extraction process, which was cross-checked for consistency by another author who resolved the disagreements according to his judgment as the most experienced reviewer. The extracted data encompassed the characteristics of clinical practice guidelines and staging recommendations.

### Quality assessment

AGREE II stands out as the most suitable instrument for assessing the quality of clinical practice guidelines ^10^. It comprises 23 items categorized into six fundamental domains, along with an overall evaluation of the utility of the recommendation:

1. *Scope and purpose:* This domain pertains to the overall objective of the guideline, evaluating whether it clearly delineates the primary goals, clinical inquiries, health facets, and target demographic.
2. *Stakeholder involvement:* This criterion determines whether the guideline was formulated by relevant stakeholders, represents the perspectives of the target demographic, and caters to users across various professions and whether the intended users are explicitly identified.
3. *Rigor of development:* This domain evaluates the thoroughness of the systematic methodologies employed for data collection, including recommendations, formulations, and the assessment of positive and negative health outcomes. Moreover, it scrutinizes the robustness and constraints of the body of evidence, the expert and external peer review process, and the guideline update protocols.
4. *Clarity of presentation:* This principle examines the clarity of the recommendations regarding language and organization as well as the ease of identifying key recommendations.
5. *Applicability:* This criterion pertains to the factors influencing guideline implementation, including barriers and facilitators, strategies for adoption enhancement, and potential resource or cost implications. In addition, it evaluates whether the guideline establishes criteria for monitoring or auditing adherence to recommendations.
6. *Editorial independence:* This domain focuses on identifying conflicts of interest or ensuring independent guideline development, which should be disclosed and managed.

Hence, two trained independent reviewers (with previous experience in applying this tool) evaluated the methodological quality of clinical guidelines using the validated AGREE II instrument. The reviewers referred to the user manual for scoring guidelines and individually completed an Excel^®^-based database to evaluate each guideline item on a scale of 1 to 7. Domain scores are calculated by summing up all the scores of the individual items in a domain and by scaling the total as a percentage of the maximum possible score for that domain, as explained in the manual ^11^.

### Statistical analysis

We performed a descriptive examination of the data. The computation of all domain scores involved aggregating the individual score per item within each domain and converting the total into a standardized percentage of the maximum possible score. The AGREE II consortium abstains from recommending a predefined quality threshold score for delineating high or low quality ^9^^,^^10^. In the present study, threshold scores of higher than 60% for the rigor of development (domain 3) and 60% in a minimum of two other domains were selected as the quality benchmark, drawing upon threshold scores documented in previous guideline assessments ^14^^-^^17^. The final score table was overlaid with colors in a heat map (≤ 25%, 26-50%, 5175%, 76-90%, and ≥ 90%) to facilitate visual comparison and interpretation, according to previous work ^17^. In addition, we conducted a narrative evaluation of the staging recommendations and evidence mapping. The study review and protocol were not registered.

## Results

### Clinical practice guideline characteristics

**Figure 1:**
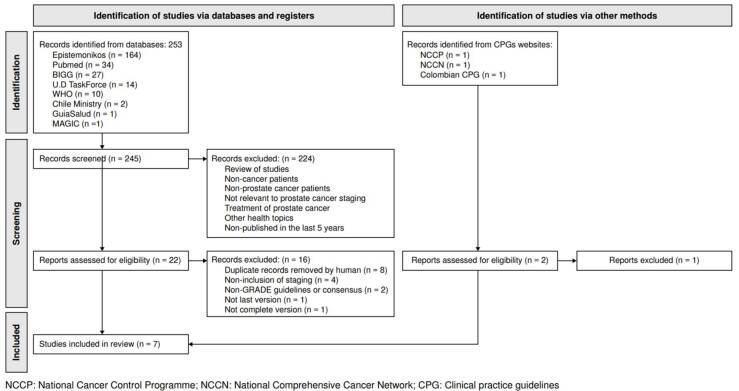
PRISMA flow chart

The examination of various databases and additional resources yielded 253 records. After screening the titles and abstracts and removing duplicates, 24 complete articles were evaluated for suitability. Seven guidelines met the eligibility criteria and were included (figure 1). The rationales for exclusion (n = 17) are shown in figure 1. Among the seven guidelines included, two were issued in the United States by the American Society of Clinical Oncology (ASCO) and the American Urological Association (AUA). The remaining five guidelines were from: the United Kingdom (n = 2), developed by the National Institute for Health and Care Excellence (NICE); Europe (n = 2), published by the National Cancer Control Programme (NCCP), the European Society for Medical Oncology (ESMO), and the European Association of Urology (EAU), and Colombia (n = 1), reported by the Colombian *Ministerio de Salud y Protección Social*. A detailed overview of the fundamental characteristics and evidence mapping of the seven clinical practice guidelines is shown in table 1.

**Table 1:** Evidence mapping (characteristics of included clinical practice guidelines)

Organization	Title	Country/ Region	Year	Methods	Scope			
					Staging	Diagnosis	Treatment	Follow-up
ASCO ^18^	Clinically Localized Prostate Cancer: ASCO Clinical Practice Guideline Endorsement of the American Urological Association, American Society for Radiation Oncology, and Society of Urologic Oncology Guideline	USA	2018	GRADE evidence	X		X	
ESMO ^19^	Prostate Cancer: ESMO Clinical Practice Guidelines for Diagnosis, Treatment, and Follow-up	Europe	2020	GRADE evidence	X	X	X	X
NICE ^20^	Prostate Cancer: Diagnosis and Management NICE guideline	UK	2021	GRADE evidence	X	X	X	X
AUA ^21^	Clinically Localized Prostate Cancer: AUA/ASTRO Guideline 2022 Endorsed by SUO	USA	2022	GRADE evidence	X		X	X
NCCP ^22^	Diagnosis and Staging of Patients with Prostate Cancer: National Clinical Guideline	UK	2022	GRADE evidence	X	X		
EAU ^7^	EAU-EANM-ESTRO-ESUR-ISUP-SIOG Guidelines on Prostate Cancer	Europe	2023	GRADE evidence	X	X	X	X
Colombian CPG ^24^	Actualización de la guía de práctica clínica (GPC) para la detección temprana, diagnóstico, tratamiento integral, seguimiento y rehabilitación de cáncer de próstata	Colombia	2023	GRADE evidence	X	X	X	X

ASCO: American Society of Clinical Oncology; ESMO: European Society for Medical Oncology; NICE: National Institute for Health and Care Excellence; AUA: American Urological Association; NCCP: National Cancer Control Programme; EAU: European Association of Urology; ASTRO: American Society for Therapeutic Radiology and Oncology; SUO: Society of Urologic Oncology; ESUR: European Society of Urogenital Radiology; EANM: European Association of Nuclear Medicine; ESTRO: European Society for Radiotherapy & Oncology; SOIG: Society of Geriatric Oncology; ISUP: International Society of Urological Pathology; GRADE: Grading of Recommendations, Assessment, Development and Evaluation

### AGREE II quality analysis

#### Scope and purpose

The Colombian guideline ^23^ had the highest score, meeting 95.23% of the criteria, followed by the ASCO guideline ^18^ with 90.47%; the ESMO guideline ^19^ only reached 23.8%.

#### Stakeholder involvement

The NICE guideline ^19^ had the highest score, meeting 88.09% of the criteria, followed by the Colombian guideline with 85.71% ^23^ and the ASCO guideline with 66.66% ^18^. Conversely, the remaining three guidelines ^8^^,^^20^^,^^21^ displayed limited consideration for the perspectives of the target population (patients, general public, etc.), resulting in scores below 50%.

#### Rigor of development

The NICE guideline ^19^ had the highest score, meeting 72.32% of the criteria. Most guidelines provided moderate information regarding the body of evidence, with individual scores ranging from 42.85 to 72.32% and an average of 60.07%. Three clinical practice guidelines ^18^^,^^21^^,^^23^ failed to outline explicit procedures for guideline updates.

#### Clarity of presentation

It had the highest mean score (71.59%), with individual scores ranging from 53.57% to 85.71%. The Colombian guideline ^23^ had the highest score, meeting 85.71% of the criteria. This indicates that all the guidelines included demonstrated adequate performance in terms of presentation quality and recommendation clarity.

#### Applicability

Across all guidelines, the AGREE II scores were significantly lower (mean score of 45.15%), except for the NICE guideline ^20^, which met 78.57% of the criteria. Three entities (ASCO, ESMO, and AUA/ASTRO) had a low score of 25% or less ^18^^,^^19^^,^^21^.

#### Editorial independence

The Colombian guideline ^23^ achieved the highest score with 85.71%; in contrast, the EAU guideline ^7^ paid limited attention to the impact of the content and disclosure of competing interests among the guideline development team members, resulting in a low score of 39.28%.

#### Summary of recommendations and levels of evidence

Five guidelines met the AGREE II threshold scores, all-encompassing staging and diagnostic suggestions, with four incorporating disease-specific treatment recommendations. The guidelines regarding prostate cancer staging, evidence level, and recommendation strength are presented in detail in table 2. All guidelines reached a consensus that imaging is not recommended for low-risk patients. Conversely, for intermediate- and high- risk categories, significant discrepancies were observed. Especially in the intermediate-risk group, some guidelines subdivided this category into favorable and unfavorable risks, dictating the necessity for imaging ^7^^,^^18^^,^^23^ or the lack thereof ^20^^,^^21^. As regards the high-risk group, five guidelines ^7^^,^^18^^,^^19^^,^^21^^,^^23^ advocated for metastatic screening, including abdominopelvic cross-sectional imaging and bone scans at a minimum. Furthermore, variations were observed in the novel imaging techniques, such as PSMA PET/CT, already incorporated by four development teams ^7^^,^^21^^-^^23^. A detailed overview of the quality analysis results is presented in table 3.

**Table 2:** Summary of prostate cancer staging recommendations and levels of evidence

CPG	Level of evidence	Strength of recommendation	Recommendation of prostate cancer staging
ASCO ^18^	High, moderate, low, very low)	Strong, moderate, weak	- “Clinicians should not perform abdominopelvic computed tomography or routine bone scans in the staging of asymptomatic very-low- or low-risk localized prostate cancer patients.” (Strong recommendation; evidence level: grade C)
			- “Clinicians should consider staging unfavorable intermediate-risk localized prostate cancer patients with crosssectional imaging (computed tomography or magnetic resonance imaging) and bone scan.” (Expert opinion)
			- “Clinicians should stage high-risk localized prostate cancer patients with cross-sectional imaging (computed tomography or magnetic resonance imaging) and bone scan.” (Clinical principle)
ESMO ^19^	IDSA-USPHS (I, II, III, IV, V)	Strong positive (A) Weak (B and D) Strong negative (E)	- “A localized disease should be classified as low, intermediate, or high risk as a guide to prognosis and therapy.” (III, A)
			- “Patients with intermediate-risk disease should be staged for metastases using computed tomography or magnetic resonance imaging of abdomen and pelvis; and bone scan.” (III, B)
			- “Patients with high-risk disease should be staged for metastases using computed tomography of chest, abdomen, and pelvis; and bone scan.” (III, B)
NICE ^20^	Low quality, very low quality, no evidence	Favor or against	- “Consider computed tomography for people with histologically proven prostate cancer for whom magnetic resonance imaging is contraindicated if knowledge of the T or N stage could affect management.”
			- “Urological cancer multidisciplinary teams should assign a risk category to all people with newly diagnosed localized or locally advanced prostate cancer.”
			- “Do not routinely offer isotope bone scans to people with Cambridge Prognostic Group 1 or 2 localized prostate cancer.”
AUA ^21^	Strength and grade (A, high; B, moderate; C, very low)	Strong, moderate, conditional, clinical principle, expert opinion	- “Clinicians should not routinely perform abdominopelvic computed tomography, or bone scan in asymptomatic patients with low- or intermediate-risk prostate cancer.” (Expert opinion)
			- “Clinicians should obtain a bone scan and either pelvic multi-parametric magnetic resonance imaging or computed tomography for patients with high-risk prostate cancer.” (Strong recommendation; evidence level: grade B)
			- “In patients with prostate cancer at a high risk for metastatic disease with negative conventional imaging, clinicians may obtain molecular imaging to evaluate for metastases.” (Expert opinion)
NCCP ^22^	High, moderate, low	Strong, weak	- “In men with favorable intermediate-risk* prostate cancer who have had a pre-biopsy magnetic resonance imaging, the use of further staging scans is not recommended.” (Quality of evidence: low; grade of recommendation: strong)
			- “In men with unfavorable intermediate-risk** prostate cancer who have had a pre-biopsy magnetic resonance imaging, the routine use of further staging scans is not recommended.” (Quality of evidence: low; grade of recommendation: weak)
			- “Is not recommended for primary staging of low-risk prostate cancer patients.” (Quality of evidence: moderate; grade of recommendation: strong)
			- PSMA PET-CT should be considered for primary staging in high-risk*** prostate cancer patients who are suitable for definitive treatment.” (Quality of evidence: moderate; grade of recommendation: strong)
			*Favorable intermediate risk is defined as having all of the following: one intermediate risk factor (cT2b-cT2c, grade group 2 or 3, PSA = 10-20 μg/L), Grade group 1 or 2, and < 50% biopsy cores positive for cancer (e.g., < 6 of 12 cores).
			**Unfavorable intermediate risk is defined as having one or more of the following: two or three intermediate risk factors (cT2b-cT2c, Grade group 2 or 3, PSA = 10-20 μg/L), Grade group 3, ≥ 50% biopsy cores positive for cancer (e.g. ≥ *6 of 12 cores)*.
			***High risk is defined as having no very-high-risk features and having exactly one high-risk feature: cT3a or grade group 4 or grade group 5 or PSA > 20 μg/L.
EAU ^7^	Oxford (1a-c, 2a-c, 3a-b, 4, 5)	Strong, weak	- “Any risk group staging: use pre-biopsy magnetic resonance imaging for local staging information.” (Grade of recommendation: weak)
			- “Treatment should not be changed based on PSMA PET/CT findings in view of current available data.” (Grade of recommendation: strong)
			- “Low-risk localized disease: Do not use additional imaging for staging purposes.” (Grade of recommendation: strong)
			- “Intermediate-risk disease: In ISUP grade 3, include at least cross-sectional abdominopelvic imaging and a bone scan for metastatic screening.” (Grade of recommendation: weak)
			- “High-risk localized disease/locally advanced disease: perform metastatic screening including at least crosssectional abdominopelvic imaging and a bone scan.” (Grade of recommendation: strong)
			- “When using PSMA PET-CT or whole-body magnetic resonance imaging to increase sensitivity, be aware of the lack of outcome data of subsequent treatment changes.” (Grade of recommendation: strong, 1b)
Colombian CPG ^24^	High, moderate, low, very low	Strong in favor, conditional in favor, conditional against, strong against	- “It is recommended not to use extension imaging in patients with low-risk localized prostate cancer.”
			- “It is recommended to use bone scintigraphy and soft tissue imaging (according to availability) as an extension study in patients diagnosed with intermediate-risk prostate cancer and unfavorable criteria.”
			- “It is recommended for high-risk patients, staging with PSMA PET/CT. In case of unavailability/opportunity, perform bone scintigraphy and conventional soft tissue imaging”.

CPG: Clinical practice guidelines; PSMA PET-CT: Prostate-specific membrane antigen evaluated by positron emission tomography/computed tomography; PSA: Prostate-specific antigen; ASCO: American Society of Clinical Oncology; ESMO: European Society for Medical Oncology; IDSA/USPHS: Infectious Diseases Society of America/U.S. Public Health Service; NICE: National Institute for Health and Care Excellence; AUA: American Urological Association; NCCP: National Cancer Control Programme; EAU: European Association of Urology; ASTRO: American Society for Therapeutic Radiology and Oncology; SUO: Society of Urologic Oncology; ESUR: European Society of Urogenital Radiology; EANM: European Association of Nuclear Medicine; ESTRO: European Society for Radiotherapy & Oncology; SOIG: Society of Geriatric Oncology; ISUP: International Society of Urological Pathology; GRADE: Grading of Recommendations, Assessment, Development and Evaluation.

**Table 3:** Heat-map showing an overview of the final AGREE II scores on guidelines recommendations for prostate cancer patients

Domain/Guideline	Scope and purpose	Stakeholder involvement	Rigor of development	Clarity of presentation	Applicability	Editorial independence	Overall assessment	Recommendations to use
ASCO	90.47	66.66	43.75	71.42	16.07	64.28	57.14	Yes = 0; No = 0; If modified = 2
ESMO	23.8	33.33	42.85	53.57	25	46.42	42.85	Yes = 0; No = 2; If modified = 0
NICE	73.8	88.09	72.32	76.19	78.57	64.28	71.42	Yes = 1; No 0; If modified 1
AUA	71.42	33.33	64.28	71.42	21.42	71.42	71.42	Yes = 2; No = 0; If modified = 0
NCCP	88.09	52.38	65.17	71.42	67.85	57.14	71.42	Yes = 1; No = 0; If modified = 1
EAU	45.23	35.71	64.28	71.42	32.14	39.28	64.28	Yes = 1; No = 0; If modified = 1
Colombian	95.23	85.71	67.85	85.71	75	85.71	71.42	Yes = 2; No = 0; If modified = 0
Mean score (SD)	69.72 (24.33)	56.45 (22.28)	60.07 (10.92)	71.59 (8.82)	45.15 (25.37)	61.21 (14.34)	64.27 (10.10)	
								
				≤25%				
				26%-50% 26-50%				
				51%-75% 51-75%				
				76%-90% 76-90%				
				≥90%				

ASCO: American Society of Clinical Oncology; ESMO: European Society for Medical Oncology; NICE: National Institute for Health and Care Excellence; AUA: American Urological Association; NCCP: National Cancer Control Programme; EAU: European Association of Urology: SD: Standard deviation

## Discussion

Clinical practice guidelines are crucial instruments that help healthcare personnel to make informed decisions based on evidence. Urology-focused organizations acknowledge the importance of clinical practice guidelines and strive toward their creation and dissemination. Disparities in the methodological rigor of clinical practice guidelines are evident regarding the objectives, financial capacities, membership, and target audience of the involved organizations. Consequently, a thorough critique of the methodology and evidence quality underpinning clinical practice guidelines is imperative before their implementation in clinical settings. The AGREE II tool enables a comprehensive evaluation of evidence supporting clinical practice guidelines.

We evaluated the methodological quality of seven guidelines for prostate cancer staging. Five guidelines (issued by the NICE, AUA, NCCP, EAU, and Colombian *Ministerio de Salud y Protección Social*) demonstrated a high standard of evidence-based methodology, indicating a meticulous framework and strict adherence to the clinical practice guideline development process. We found paramount concerns regarding the domain of applicability, followed by stakeholder involvement, corroborating previous findings of Gupta *et al*. ^24^. We prioritized aspects such as validity, implementation strategies, and impact of clinical practice guidelines. However, four guidelines failed to address the incorporation of facilitators and barriers, hindering the monitoring of recommendation adherence.

Furthermore, most guidelines lack explicit directives concerning language, structure, and formatting criteria within the fourth domain (clarity of presentation). Particular emphasis was placed on acknowledging independent guideline development and disclosing potential conflicts of interest among organizations, a factor pivotal for fostering trust. This domain achieved an average score of 61.21%, with notable variations observed across guidelines. Data collection encompassed multiple documents per organization, extending beyond the most recent guideline versions in full text. Notably, the NCCN guideline ^25^ played a fundamental role in furnishing a comprehensive framework for diagnosing, treating, and monitoring patients with prostate cancer. However, its exclusion was warranted due to the distinct methodology employed by the development group, based on the NCCN categories devised by the authors, hindering a comprehensive methodological appraisal and comparison with other guidelines.

Scarce literature exists evaluating the methodology of prostate cancer guidelines, with limited use of assessment tools. Gupta *et al*. assessed 13 clinical practice guidelines for the treatment of localized prostate cancer, published from 1999 to 2014, excluding from analysis the early detection, screening, diagnosis, or staging of prostate cancer (the focus of this study). Clarity of presentation had the highest median score (87.5%), followed by editorial independence (85.4%), and then scope and purpose (84.7%); applicability had the lowest score (28.1%) ^24^. This study assessed published clinical practice guidelines about the treatment of localized prostate cancer to evaluate rigor, applicability, and transparency of the recommendations. A similar study focusing on screening four prostate cancer guidelines in the United States yielded analogous outcomes, with optimal scores in domains 1 and 4 and the least favorable score in domain 2 ^26^. The Capacity Enhancement Program in Ontario used to provide training and quality assessment of guidelines with the AGREE II tool. This program scored 46 clinical practice guidelines related to prostate cancer promotion, screening, diagnosis, and staging, revealing substantial variability across the following domains: applicability (mean = 25%; range = 4-73%), editorial independence (mean = 31%; range = 0-94%), and clarity of presentation (mean = 67%; range = 27-97%) ^27^.

Our findings are consistent with previous reviews, indicating less diversity in evaluated areas across different clinical practice guidelines. This trend is likely because of the ongoing updates and shifts toward higher- quality standards over time. Consequently, the methodological quality of clinical practice guidelines developed by diverse entities exhibits significant heterogeneity. The design of more robust and superior guidelines is crucial to facilitate urologists in making well-informed clinical decisions based on the most reliable evidence available.

Recent guideline updates feature staging recommendations from various groups working on clinical practice guidelines, with a notable inclusion of the PSMA PET/CT in high-risk patients instead of traditional imaging methods. This advancement has been proposed by the EAU, AUA, and NCCP guidelines, and is integrated into the 2023 Colombian guideline update ^23^. PSMA PET/CT exhibits superior discriminatory capabilities in assessing extracapsular involvement, nodules, and distant metastases compared to conventional images ^7^. Previous studies have demonstrated its increased sensitivity in detecting nodal and bone metastases compared with bone scans and abdominopelvic computed tomographies ^28^. Considering its efficacy, the use of PSMA PET/CT could be contemplated in the initial staging of high- risk prostate cancer. However, further prospective research is imperative to establish its impact on survival rates, prognosis, and optimal management strategies ^7^^,^^29^.

Our study has certain limitations. First, the AGREE II tool solely permits the appraisal of guidelines’ methodological quality and applicability. The evaluation of methodological quality is inherently subjective. The tool lacks predefined thresholds to differentiate between high and low quality, as it was developed by independent assessors. Second, reliance on cutoff scores set by previous studies ^15^^-^^17^ may lead to varied result interpretations. Also, the protocol was not previously registered. Some steps were not performed in duplicate but were reviewed by a second author, which could imply a risk of measurement bias.

To conclude, our comprehensive examination revealed an increasing need to enhance the methodology and standard of clinical practice guidelines. Thus far, guidelines related to prostate cancer staging -issued by various institutions- exhibit a significant heterogeneity in methodological quality, failing to meet current standards across multiple criteria. Primary deficiencies are commonly observed in the domains of “applicability” and “engagement of stakeholders,” limiting the use of guidelines by healthcare personnel. In addition, this study highlights the need for more stringent and superior-quality clinical practice guidelines.
